# Bitumen Foaming Optimisation Process on the Basis of Rheological Properties

**DOI:** 10.3390/ma11101854

**Published:** 2018-09-28

**Authors:** Marek Iwański, Grzegorz Mazurek, Przemysław Buczyński

**Affiliations:** Department of Transportation Engineering, Faculty of Civil Engineering and Architecture, Kielce University of Technology, Al. Tysiąclecia Państwa Polskiego 7, 25-314 Kielce, Poland; iwanski@tu.kielce.pl (M.I.); p.buczynski@tu.kielce.pl (P.B.)

**Keywords:** foamed bitumen, Fisher-Tropsch wax, design of experiment, bitumen rheology, foam decay parameters

## Abstract

This article discusses the results of bitumen foam properties optimisation with respect to three factors: air pressure, bitumen temperature and amount of water. The test materials were unmodified bitumen 50/70 and bitumen 50/70 modified with 2.5% synthetic wax. The experiment was designed according to the 3^(3−1)^ fractional factorial design. The distribution of parameters of bitumen foam were measured with the authors’ original apparatus using a laser beam. This measurement method increased the accuracy of maximum expansion ratio (ER) and half-life (HL) estimation. Based on HL and ER results, it was found that the foaming process increased bitumen stiffness due to the dynamic ageing of the bitumen. The experimental design allows more effective control over the properties of foamed bitumen with respect to its intended use. The presence of synthetic wax extended the half-life of the bitumen foam.

## 1. Introduction

One of the important factors for obtaining durable bituminous mixtures is the use of bitumen with a viscosity that will provide the mix with high in-service resistance to permanent deformation that correlates with high stiffness modulus and with adequate workability in the manufacturing process [1,2]. Foamed bitumen technology, aimed at reducing bitumen viscosity, was initially applied to the stabilisation of soils and road subgrades in which bitumen foam was used as a binder [3,4]. Bitumen foam exhibited poor aggregate coating ability during the first stage of its application to pavement upper layers. The situation changed with the introduction of half-warm mixtures in which the aggregates were pre-heated. Then, the workability of the mixture increased due to the drop in viscosity of H-WMA (half worm mix asphalt). As a result, the parameters of the H-WMA mixture were comparable to the traditional HMA technology (hot mix asphalt) [5,6]. Research results reported in [7,8] indicate that foam expansion and its stability are dependent on the proportion of water used in the foaming process. It affects the size and distribution of the foam bubbles, thereby the shape of the foam decay curve [9] and the foaming index (FI) need to be determined [7]. This approach, however, is controversial due to a complex shape of the foam decay curve and the fact that a decrease in half-life (HL) may not correspond to an increase in the expansion ratio (ER). Saleh [10] demonstrated that bitumen viscosity could be a more useful measure of bitumen foam quality than the measurement of only two parameters, ER and HL. Viscosity can be reduced with the application of zeolites [11] or of synthetic waxes that improve foaming parameters by stabilising foam decay [12], as does increasing the asphaltene fractions [13]. Chemical foam-enhancing agents are also used to improve foam stability [9].

Foam distribution evaluation is strongly influenced by the accuracy of measurement. Generally, ER and HL based assessment does not provide exhaustive information about to what extent the foaming process affects the changes in bitumen rheology and chemical composition [14]. ER and HL parameters depend on temperature, water quantity, and pressure used in the foaming process. Usually only the water content is controlled. The effect of other parameters on the quality of foam depends on the type of bitumen used. The process of foaming causes a rapid change of water into vapour in hot bitumen. The presence of water vapour can contribute to a significant increase in bitumen stiffness. This hardening of the bitumen is a consequence of the distilling water vapour action on petroleum oils [2]. The process is similar to that of gudron hydrocracking in a vacuum tower. Acceleration of the ageing process was also observed in the simulation of bitumen ageing in the presence of water [15]. High temperatures with high water vapour pressure may cause the foamed bitumen to reach a high degree of stiffness over time. Information on optimal foaming, accounting for all aspects of the foaming process, ageing and minimum stiffness of H-WMA mixtures during the service period is scarce in the literature.

This article discusses the influence of foaming process quality on rheological properties of bitumen, considering viscosity changes after foaming and bitumen modification with synthetic waxes. The optimum solutions with several criteria accounted for will contribute to broadening the knowledge of the bitumen foaming process.

## 2. Materials and Methods

### 2.1. Bitumen

Paving bitumen 50/70, assigned to the sol-gel rheological type, was used in the tests without modification and modified with 2.5% Fischer-Tropsch synthetic wax (FT wax), denoted as 50/70 M [16,17]. The amount of the synthetic wax incorporated into neat bitumen was optimised [18]. Basic standard parameters determination was supplemented with the measurement of stiffness modulus G*, phase angle δ, dynamic viscosity η and zero shear viscosity η_ZSV_. The additional rheological parameters were determined at 60 °C. The results are summarised in Table 1.

### 2.2. Sampling

Foamed bitumen samples were collected in separate glass containers. The number of containers followed the experimental design assumptions, with necessary replication. To maintain the properties of the bitumen after foam collapse, the adopted protocol assumed cooling the samples to the temperature of −10 °C 15 min after foaming [14,19]. The samples were stored under these conditions until the rheological parameter determination started.

### 2.3. Dynamic Viscosity

Viscometer Rheotest 4.1 was used in compliance with the relevant standard [20]. Dynamic viscosity was determined at 60 °C for two levels of shear rate, i.e., for dynamic viscosity of the Newton’s law state, $\dot{\gamma }\;$= 1 s^−1^ (experimental) and for zero shear viscosity η_ZSV_ determined at a shear rate of 0.0032 s^−1^ [21].

### 2.4. Rheological Tests in Oscillation

Rheological tests for the complex shear modulus G*and phase angle δ were performed with a cone-plate (25 mm) viscometer with a gap of 150 µm between the measuring systems, operated in forced oscillation mode [22,23]. The stress amplitude used in the tests was 40 Pa (LVE) and the test temperatures were 40 °C and 60 °C. The frequency range was between 0.1 Hz and 10 Hz. The linear viscoelastic range stress was established using procedure and model found in SHRP-A-370 [24].

### 2.5. Experimental Design 3^(3−1)^

Three independent factors were considered. These factors/variables controlled the foaming process. Typically, the optimal temperature for foaming is greater than 140 °C. However, using a modifier such as synthetic wax allows decreasing the bitumen viscosity; therefore, it was assumed that the foam could be manufactured below that temperature limit. The main purpose of this study was also to conduct an experiment at low temperature regardless of the final result. To perform an optimisation, this experiment was carried out beyond a typical apparatus setting. The factors represented the settings of the apparatus, which in this case was the laboratory-scale bitumen foamed plant WBL-10S. The experiment was performed using the following WLB-10S setting values (independent factors):

- Air pressure: 100 kPa, 400 kPa, and 700 kPa;
- Bitumen temperature: 110 °C, 145 °C, and 180 °C; and
- Water content: 1%, 2.5%, and 5%.

A detailed description of the 3^(3−1)^ fractional factorial design is given in [25,26]. This fractional factorial experiment plan is a special case of full factorial plan. Due to the high labour intensity, the effects of interaction between factors (interactions) have been omitted. The fractional factorial design plan 3^k−p^ was built (assuming that the corresponding synergy effects are equal to zero) by comparing the p columns of interactions in the full plan 3^3^ to the columns of independent variables. The number of N experiments, forming this plan, should be greater than the number k + 1 specifying the number of regression coefficients. Therefore, creating plan for k independent variables, the p was set in such way that N = 3k − p > k + 1. According to this, for this design, no assessment of variability induced by interactions between independent variables was performed. In this study, the design was developed by comparing interaction columns to the columns of new independent variables, as a result of which the number of factor combinations was reduced from 27 to 9 and only the most important part of the full design was run. This approach is a trade-off between maximisation of information from the model approximation and the effort made when the full design is used. The design was randomised within the blocks to reduce the systemic error appearing during the sequential sampling and determining the parameters. Table 2 compiles the WLB-10S setting combinations.

This design required variables to be coded. For example, 400-145-2.5 means that the bitumen foaming process was performed at 400 Pa, 145 °C and 2.5% foaming water. Adoption of the model had to meet the condition of the number of factor combinations being greater than the number of model parameters sought plus one. A second order polynomial model without interaction was adopted as an appropriate mathematical function. To determine the measured variable (Y), seven unknowns included in the regression equation were estimated, which were essentially the second order polynomial without interaction [25] (Equation (1)):

(1) $$\;Y=b_{o}+\overset{k}{\underset{i=1}{\sum }}b_{i}\cdot x_{i}+\overset{k}{\underset{i=1}{\sum }}b_{i}\cdot x_{i}{}^{2}\;$$

where: $x_{i}$ is an independent variable (laboratory-scale bitumen foamed plant setting), $b_{i}$ represents experimental parameters of regression, Y is a dependent variable, and k is the number of unknown regression parameters (seven in the experiment).

The model was used to predict both foaming parameters and rheological parameters of bitumen after foaming. The quality of fit of the objective function mathematical model to the experimental results was determined using the coefficient of determination R^2^ and the declared value of the root mean square error (RMSE).

## 3. New Testing Method for Foam Decay Determination 

### 3.1. Laser Measurement of HL and ER

**Figure 1:**
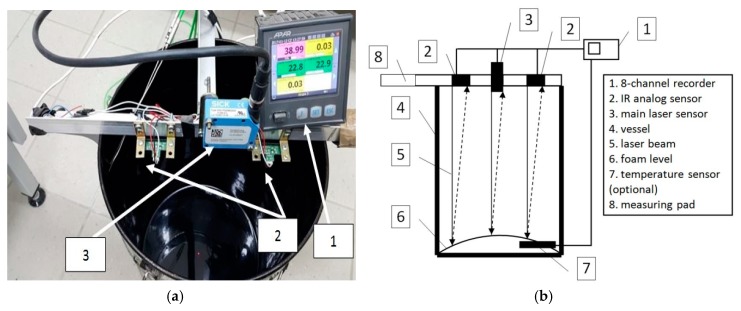
Original device for measuring bitumen foaming parameters (1, recorder; 2, IR analogue sensors; 3, laser sensor): (**a**) perspective frontal view; and (**b**) schematic diagram.

To minimise the influence of the observer on the results of the foaming parameters, i.e., on the maximum expansion (ER) and the half-life of the foam (HL), the prototype digital measuring device was used. The device is primarily based on the use of a highly accurate laser sensor, similar to that used by Newcomb et al. [27], for non-contact measurement of displacement based on the principle of optical triangulation. In [28], a laser was also used as the basic tool for measuring the change in the volume of asphalt foam. The laser diode illuminates a point on the surface to be measured. The light reflected from this place is directed to the photosensitive matrix, where it is processed in real time. The declared accuracy of the distance measurement for the 50 mm range is ±15 μm. The unit with the digital recorder and its diagram are shown in Figure 1.

The device was constructed following the findings reported in the literature. Arguelles et al. ([14] based on [29]) stated that the measurement results for bitumen foaming parameters depend strongly on the operator. The device consisted of a centrally located high precision laser sensor “3”. At half the distance between the main sensor “3” and the container sideline, two IR analogue sensors “2” with accuracy of ±3 mm were mounted to verify the operation of the main sensor “3”. The results were recorded using the eight-channel recorder “1” with 1 s measurement interval. Typically, the measurement of the foaming parameters is carried out by means of a measuring rod together with a calibrated container (for ER) and a stopwatch (for HL). The calibrated measuring rod has a resolution of ER quintuple. The distance laser sensor has a resolution of ±5 μm for the 50 mm range. To compare the accuracy of expansion measurement, uncertainty was determined based on a number of relevant and known factors affecting the ER measurement. The preliminary uncertainty assessment followed the generalised pattern of the sum of systematic errors [30] of expanded uncertainty (Equation (2)):

(2) $$\;U\left(y\right)=k\cdot \sqrt{\overset{m}{\underset{j=1}{\sum }}{\left[\frac{\mathrm{df}\left(x\right)}{{\mathrm{dx}}_{j}}\cdot u\left(x_{j}\right)\right]}^{2}}\;$$

where: U(y) is the expanded uncertainty of variable y, $u\left(x_{j}\right)$ is the standard component uncertainty (partial uncertainty), and k is the coverage factor associated with the confidence interval.

The analysis did not account for random error because the database containing the repeatability results for both methods with respect to one bitumen type and one combination of device settings did not reach the result close to 30 runs. Table 3 compiles expanded uncertainties of both methods, including the distribution shapes of systematic error results.

Note that the ER measurement uncertainty from the prototype device is a factor of 6 times smaller than the result obtained from the measuring rod. Time registration (for HL) is far more accurate. In this case, uncertainty related to the operator’s perception is eliminated.

### 3.2. Bitumen Foam Distribution Model

A single bitumen sample for a given settings combination (combination of foaming parameters) of the WLB-10S plant was fixed at 500 g. The bitumen flow rate was set at 100 g/s. Continuous measurement of the ER using the laser device required recording the height of the foam in the measuring device and its corresponding expansion time. Precise estimation of foaming parameters, ER and HL, needed to find a mathematical relationship between them. Based on the mathematical description of bitumen foaming reported in the literature [7,9], the sigmoid function was proposed. The general form of this function is used in pharmacological experiments and also in modelling the bitumen viscosity versus shear rate. The shape of this function coincides with observations of changes in the volume of bitumen foam. After some mathematical transformations, this model was adopted for modelling the bitumen foam decay. The foam bubbles rapidly collapse during the first five seconds, making it impossible to measure properly. Therefore, the behaviour of the foam during the first 5 s was determined by the extrapolation of the assumed function of the model. Its character is represented in Equations (3) and (4):

(3) $$\;\mathrm{ER}\left(t\right)=1+\frac{{\mathrm{ER}}_{\max}}{1+{\left(\frac{t}{\mathrm{HL}}\right)}^{c}}\;$$

(4) $$\;{\mathrm{ER}}_{\max}=\frac{\max\left(b\left(t\right)\right)}{a}\;$$

where ER(t) is the calculated expansion ratio vs. time, a is the height of 500 g of non-foamed bitumen in the container (a constant value), b(t) is the distance between the foamed bitumen upper surface and the sensor (changing over time), HL is the model half-life, t is the measurement time, c is the ratio defining the foam decay rate (the ratio of changing HL vs. time), and ${\mathrm{ER}}_{\max}$ is the maximum of expansion ratio (ER).

The choice of this model was dictated by the fact that, by means of a small number of parameters, it was possible to satisfactorily characterise how quickly the volume of foamed bitumen was changed. Based on the conducted simulation and considering the resolution of the recording device, it was found that Equation (3) best represented the expansion of bitumen foam and its stability. Nevertheless, such model is a hypothesis, which does not exclude the use of more advanced mathematical models. To determine model parameters in Equation (3), the experiment was performed twice. In the case of a large discrepancy, a third replication was used. The model does not exhaust all the behaviours of bitumen foam, but in the case of the bitumens being analysed the matching of functions to the results was very high. In the cases where calculated HL is extremely low, below 1 s, either the foaming effect in bitumen did not take place or was hard to estimate. Unusually, the low value of HL was related to either low temperature (110 °C) or low pressure (100 kPa). Regardless of a low HL, all test results were taken for further analysis. Only in few cases the R^2^ parameter was about 0.6. In most cases, it was >0.7. The results of parameter matching, the mean of the two results, of the function describing the distribution of the foaming parameters are compiled in Table 4. Additional measurements were used to determine the variance of repeatability required to calculate the significance of the parameters.

**Figure 2:**
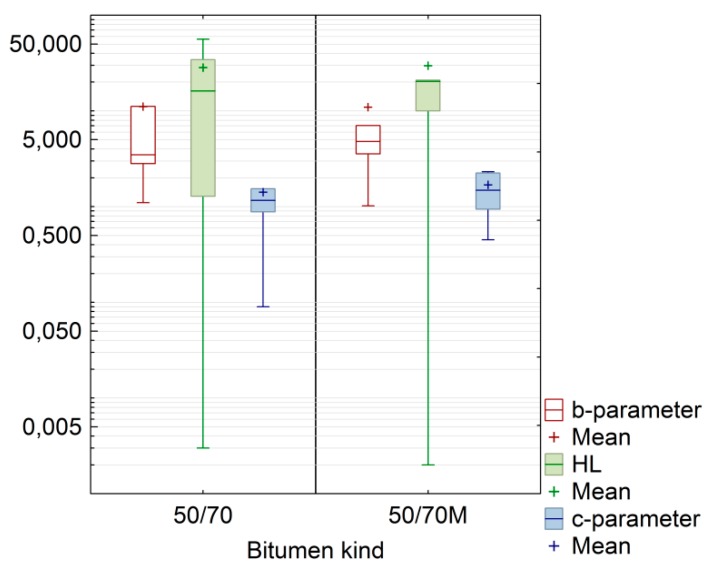
Box-and-whisker plot for the bitumen foam distribution parameters.

The set of results of the model parameters assigned in accordance with the experimental design was used to make a preliminary comparison of the foam distribution parameters for the bitumen foam degradation rate (parameter c), ER determined by the foam height in the container (parameter b) and HL. A box plot depicting 50% distribution of the population through its quartiles was used (first quartile 25% and third quartile 75%). The summarised results of the evaluation are shown in Figure 2.

**Figure 3:**
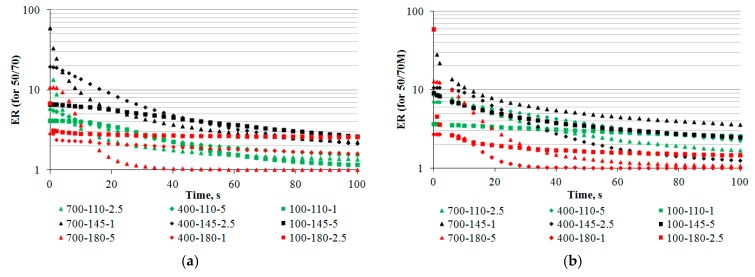
Distribution of bitumen foam according to the logit of the model (5 s resolution): (**a**) bitumen 50/70; and (**b**) bitumen 35/50 M.

Since the model parameters have different scales, they are all included in one graph using logarithmic scaling. The preliminary comparison by bitumen type was used with the median result (the line in middle of the box plot) instead of the arithmetic mean. By comparing the average value of the population to the median, bitumen 50/70 M was found to have slightly higher ER (represented by parameter b) and slightly higher HL. The foam decay rate (parameter c) of bitumen 50/70 was comparable to that of bitumen 50/70 M, but the 50/70 M data were less spread out than the 50/70 bitumen data. Longer and more gradual HL and the stable c value extends the time at which bitumen foam is suitable for use. With longer HL, the 50/70 M foam will be more suitable for H-WMA mixtures and for deep recycling with RAS. This seems to be related to the rheological properties characteristic of a given bitumen composition. A graphical interpretation of the foam distribution model against the cases imposed by the experimental design at a 5 s resolution is shown in Figure 3. To increase the legibility of the graphical representation in Figure 3, only the results from numerical simulations based on the model in Equation (3) with the parameters in Table 4 are included.

After completing the stage of estimating the ER, HL (foaming parameters), the coefficients of the assumed model in Equaiton (1) were estimated. To estimate the significance of these coefficients, two additional measurements of bitumen foam were used for case 400-145-2.5 They played role of “fictitious” additional experiments (Table 4). It was necessary to evaluate a significance of the model coefficients in Equation (1). Aforementioned polynomial equation was fitted to the history of ER and HL plots. Table 5 summarises the results for the model parameters.

**Figure 4:**
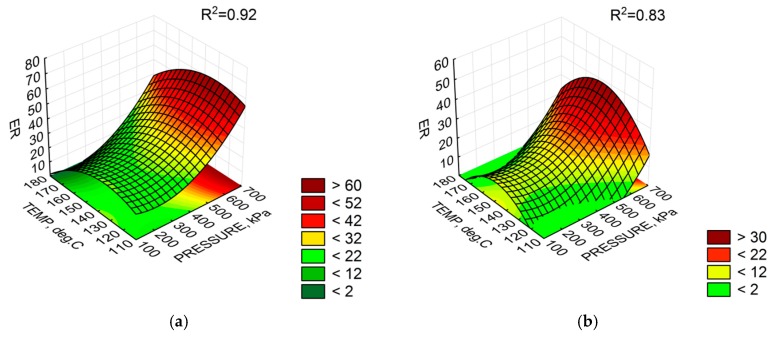

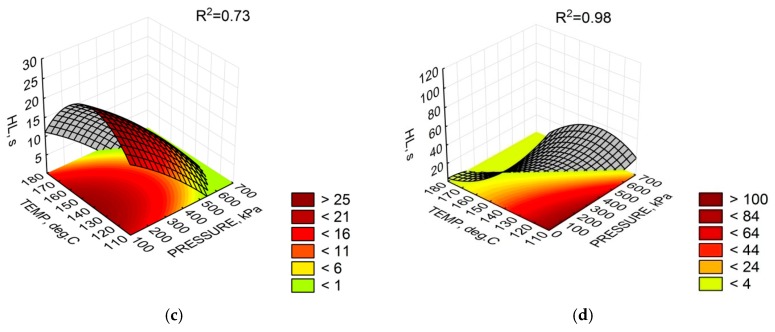
Response surface of foam parameters (2.5% water amount): (**a**) expansion ratio (ER), 50/70; (**b**) expansion ratio (ER), 50/70 M; (**c**) half-life (HL), 50/70; and (**d**) half-life (HL), 50/70 M.

It should be noted that ER variability, irrespective of the bitumen type, was affected by all independent variables, i.e., the bitumen temperature, air pressure and amount of water. As for the HL parameter, water pressure had the least effect in the model. Analysis of the quality of fitting the model to the results of ER for bitumen 50/70 revealed the estimation error ER_50/70_ = ±4.0, greater than the estimation error for bitumen 50/70 M, ER_50/70M_ = 1.3, indicating that the results would be more uniform for the FT wax modified bitumen. The polynomial model explained ER changes far better for bitumen 50/70 (R^2^ = 0.92). However, the difference was small compared to the bitumen with synthetic wax. Graphical interpretation of the foaming parameter distribution at 2.5% water dose (the middle value of the experimental design) for both bitumen types is given in Figure 4.

Analysis of the results (Figure 4a,b) shows that, at the 2.5% water content, ER was similar for both bitumen types. The highest ER parameters for bitumen 50/70 were obtained at a temperature of about 140 °C and a pressure of above 400 kPa. The increase in temperature lowered the viscosity of the bitumen to limit the formation of large air bubbles, just as high viscosity did at low temperatures. With this setting range of the WLB-10S, the low HL of the foam was inversely proportional to the results of the ER. In the case of 50/70 M bitumen, the ER maximum was reached at a temperature of approximately 145 °C and a pressure of >600 kPa. However, the ER values of bitumen 50/70 M were generally lower. As for the HL parameter of bitumen 50/70 M, the bitumen foam rapidly decayed with an interactive rise in bitumen and water pressure. This character of bitumen foam decay in the 50/70 M bitumen was probably due to the low viscosity of bitumen dispersed by the synthetic wax phase. As a result, the decrease in bitumen cohesion contributed to the rapid foam collapse. It is apparent that the 2.5% water content does not have to be the optimum amount for the bituminous types tested. The foaming process will thus change the rheological properties of the bitumen in a different manner.

## 4. Results of Rheological Parameters

### 4.1. Viscoelastic Characterisation of Bitumen Foam

**Figure 5:**
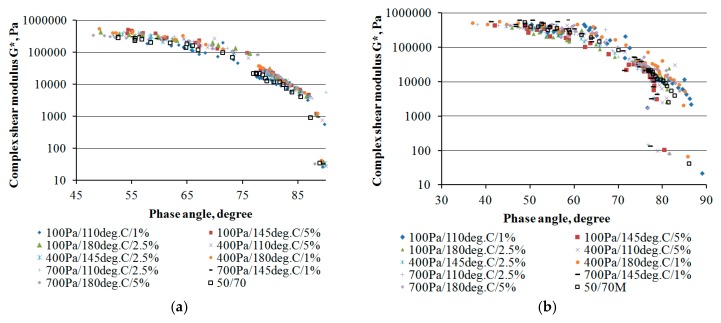
Black curve based on the data determined at 40 °C and 60 °C: (**a**) bitumen 50/70; and (**b**) bitumen 50/70 M.

The viscoelastic nature of the bitumen foam was determined by measuring the complex shear modulus G* and the phase shift angle δ in the frequency range 0.01–10 Hz and for temperatures of 40 °C and 60 °C. The Black curve makes the representation of the results combining frequency and temperature that allows the comparison of the viscoelastic nature of various bitumens. Figure 5 shows the Black curves for bitumens 50/70 and 50/70 M with different combinations of bitumen foaming programmes, and with non-foamed reference bitumens.

The phase shift angle is considered more sensitive to chemical changes in bitumen [31]. The results of the Black curve analysis for bitumen 50/70 revealed a slight shift in the results of the G* after the foaming process towards the higher phase angle values (Figure 5a) relative to the reference bitumen. This behaviour of the bitumen after the foaming process illustrates the increase in dominance of the imaginary component G’’ over the real component G’. The Black curve shifted also with respect to the vertical axis and was related to the increase in the value of the complex modulus G* after foaming relative to the reference bitumen. Such a change in the rheological nature of the bitumen after foaming indicates chemical changes in the bitumen, caused by oxidation in the bitumen ageing process [31,32]. Only the 100-110-1 variant of bitumen foaming gained a slightly more viscous character than the reference bitumen. This may be related to the large number of small air bubbles in the bitumen which, at the stress applied, cause a rapid collapse of the bitumen structure and its yielding. The presence of entrapped air bubbles probably was also responsible for the predominance of the viscous part in the absolute value of the complex stiffness modulus, which, in turn, could accelerate the accumulation in irreversible deformations that could arise as a result of cyclic loading in the layer made with foamed bitumen. This is because the high level of G” affects the increase in amount of dissipated energy in one loading cycle [1].

The Black curve plots for the modified material, 50/70 M, are slightly different from those of bitumen 50/70. The use of synthetic wax in the bitumen after the foaming process resulted in the shift of the foamed bitumen Black curves even further towards the smaller values of the phase angle. It can be seen that, for the curve range of high temperatures, in some cases of foamed bitumens have a lower phase angle compared to the reference bitumen (Figure 5b). This is attributed to the presence of synthetic wax crystallites in the bitumen, increasing the molecular weight and imparting a more elastic character to the bitumen, particularly at high service temperatures, in this case at 60 °C. As a result, the presence of FT wax could compensate a loss in elasticity of the bitumen after foaming caused by presence of entrapped air in bitumen. This characteristic can be particularly beneficial in mixtures produced by deep-cold recycling. For the intermediate frequency/temperature range, the curves fluctuate compared to the reference bitumen curve. Bitumen foams produced at low water pressures usually achieve lower values of complex shear modulus, whereas high temperatures promote its increase in the 50/70 M bitumen. Nevertheless, the discrepancy between the Black curve plots of foamed and reference bitumens can be attributed to entrapped air (amount of noncollapsed bubbles), presence of wax crystallites and chemical changes occurring in FT wax modified bitumen during foaming. It should also be noted that the presence of synthetic wax alters the rheological character of the bitumen to be considerably more elastic—this characteristic is more pronounced than in the foamed 50/70 bitumen.

### 4.2. Effect of Foaming on Stiffness Modulus G*/sin(δ)

**Figure 6:**
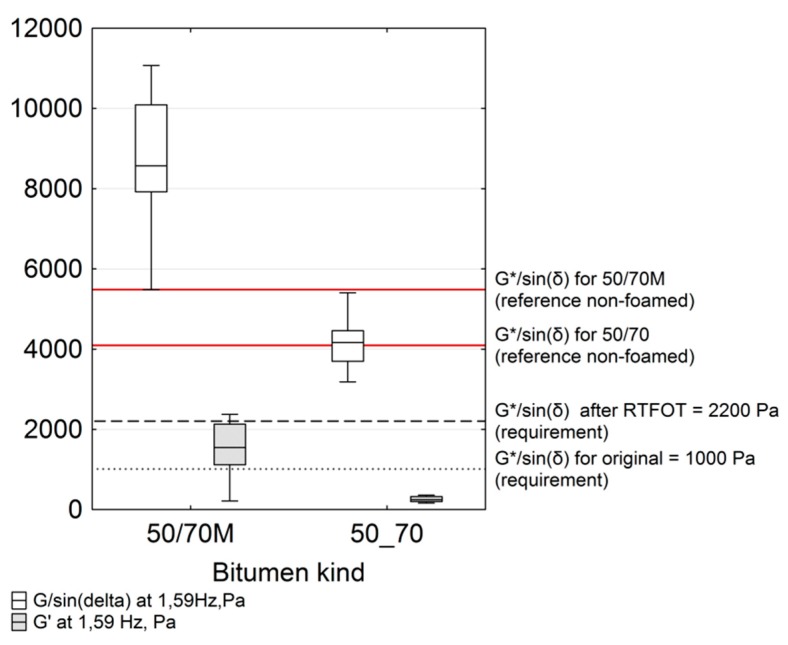
Box plot for G*/sin(δ) and the elastic component of the complex modulus at frequency 1.59 Hz at 60 °C.

The rutting resistance index G*/sin(δ) [23], developed by the Strategic Highway Research Program (SHRP) launched in 1987, measured at 60 °C and at 1.59 Hz characterises the imaginary part of the bitumen complex susceptibility [33,34]. It was introduced to eliminate the bitumens with excessively high susceptibility in terms of the resistance of bituminous mixtures to rutting [1]. Despite criticisms of its use for assessing the susceptibility of polymer modified bitumens, in the case of unmodified bitumen, it has been found to be in good compliance with the MSCR test results for linear viscoelasticity [1,35], i.e., the stress of 100 Pa. The increase in G*/sin(δ) may be caused by changes in the bitumen fractional composition due to the aromatic hydrocarbon condensation process during ageing as well as due to the crystallisation of synthetic wax. As in the previous section, the results of the calculated G*/sin(δ) parameter were approximated with respect to the adopted experimental design, as shown on the box plot diagram in Figure 6. 

Analysis of the population (Figure 6) clearly indicates that the increase in G*/sin(δ) for bitumen 50/70 M after foaming compared to bitumen 50/70 is much more dynamic. The median of the unmodified foamed bitumen results is near the level of G*/sin (δ) with respect to bitumen 50/70.

Nearly all 50/70 M bitumen results are higher than those of non-foamed bitumen. The distance between the median of the results distribution and the G*/sin(δ) value are significant. Moreover, comparing the median result, the level for 50/70 M is higher than for 50/70. Kruskal–Wallis nonparametric tests show a significant difference in the assessment of the average G*/sin(δ) value, depending on the bitumen type tested. A similar significant difference was observed for the average value of elastic component, G’, of the complex modulus. FT wax crystallites present in the bitumen are responsible for the increase in the elasticity of the tested bitumens [36]. The marked G*/sin(δ) limits determined by [15] indicate that at the test temperature of 60 °C, common under Polish conditions in summer months, rutting will be significantly limited in all bitumen types tested.

On the other hand, this significant increase in bitumen stiffness at service temperatures will contribute to higher brittleness of the bitumen at low temperatures and high critical temperature (fatigue) defined by G* × sin(δ) < 5000 Pa. In addition, this bitumen stiffness will noticeably affect the stiffness of low temperature and recycled bituminous mixtures [37]. The lowest value not distant from other observations in the quartile distribution with respect to the G*/sin (δ) parameter was reached by the case denoted as 100-110-1 (pressure, Pa; foaming temperature, °C; water content, %). Regardless of the bitumen type of bitumen, the G*/sin(δ) parameter was lower than the base bitumen. This is an interesting observation because the resulting bitumen stiffness is lower than that of the base bitumen. This singularity was noticed during the analysis of the Black curves. The trapped air in the form of bitumen bubbles and the marginal effect of ageing could affect the bitumen yielding in any bitumen type. A wider discussion of this singularity will be presented when analysing the results of the bitumen hardening index.

As in the case of foam distribution analysis, also in the case of G*/sin(δ) parameter evaluation, its parameters were described by the regression model adopted in the experimental design. The results of the adjustment of experimental parameters are shown in Table 6.

**Figure 7:**
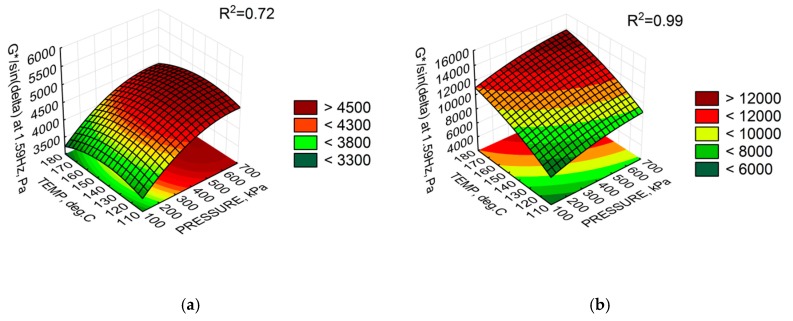
Response surface of G*/sin(δ)at the 2.5% water content: (**a**) bitumen 50/70; and (**b**) bitumen 50/70 M.

The analysis of fitting the G*/sin(δ) distribution to the objective function model assumed in the experiment shows a good fit for bitumen 50/70 M. The explanation of the G*/sin(δ) parameter variability is 99% with an average estimation error (RMSE) of ±128.3 Pa. All parameters turned out to be significant. A high sensitivity of G*/sin (δ) to slight changes in the setting of the foaming device was confirmed. As for bitumen 50/70, the model explains only 72% of the variability of this parameter. The error of estimation, ±137.3 Pa was not significantly greater than that for the modified bitumen. In the case of unmodified bitumen, 50/70, only the linear coefficient for water pressure and the non-linear temperature coefficient were found to be significant. The remaining cases were statistically insignificant. This result clearly indicates the slight sensitivity of G*/sin(δ) due to the 50/70 bitumen foaming process compared to the 50/70 M bitumen. This conclusion is supported by the analysis in Figure 6. Graphical representation of the G*/sin(δ) distribution is shown in Figure 7.

The response surfaces shown in Figure 6 indicate that the value of G*/sin(δ) is not significantly changed in the bitumen 50/70 at pressure above 600 Pa, temperatures above 140 °C and 2.5% water quantity. With doubtful *p*-value for the non-linear coefficient of temperature, the temperature effect can be assumed to be negligible and may have a constant value for the specified range. For the 50/70 M bitumen, temperature and pressure increase results in improved foaming parameters (Table 6) and a statistically significant increase in G*/sin(δ), leading to increased stiffness of the 50/70 M bitumen.

### 4.3. Effect of Foaming on the Ageing Index (IS)

**Figure 8:**
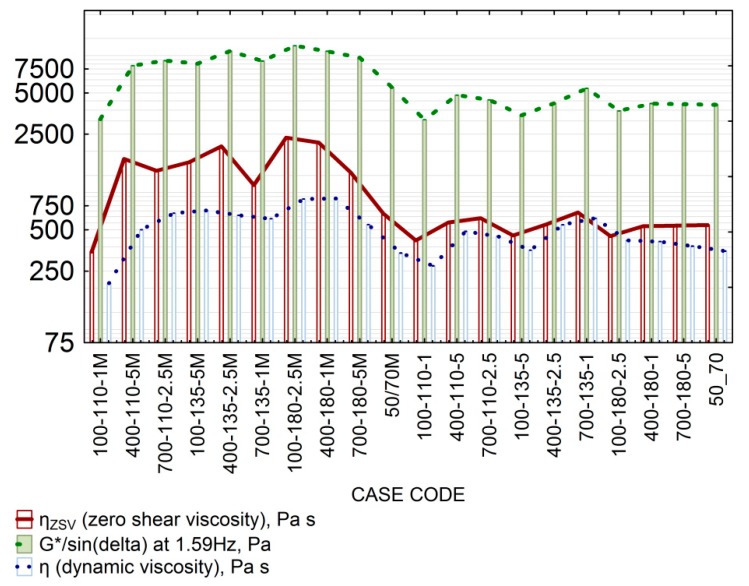
Foaming process (mean values) influence on the dynamic viscosity η, zero shear viscosity η_ZSV_ and G*/sin(δ) at 60 °C.

The observation about excessive increase in bitumen stiffness during the foaming process, probably due to bitumen ageing, was supplemented by an analysis of the dynamic viscosity parameter. Its results were used to determine the ageing index. Dynamic viscosity was determined according to Section 2.3 for bitumen before and after foaming. The dynamic viscosity results were compared with the results of zero shear viscosity, η_ZSV_. The results of G*/sin(δ) were added to the scale, as shown in Figure 8. All tests were performed at 60 °C.

Analysis of the viscosity results and the difference between dynamic viscosity (Newtonian viscosity state II) and zero shear viscosity (Newtonian viscosity state I) indicates that the dynamic viscosity difference in the Newtonian states in 50/70 is smaller than in 50/70 M. The minor difference observed indicates that the viscosity measurement temperature is close to that of the bitumen behaving like a viscous liquid. A slight increase in the dynamic viscosity and G*/sin(δ) at 60 °C relative to the reference bitumen 50/70 is a proof that bitumen after foaming is affected by ageing. In bitumen 50/70 M, the difference between dynamic and zero shear viscosities is noticeably greater, which suggests a more non-Newtonian character of 50/70 M bitumen, with a strong influence of synthetic wax crystallites on viscosity increase in intact state. However, the proportions of differences between viscosity states in terms of values were roughly the same. This result can be attributed to the presence of synthetic wax crystallites. The difference increases with increasing foaming temperature (50/70 M) and with increasing amount of water used in the foaming process. Similarly, in the process of bitumen production, excessive temperature of crude oil distillation in the presence of water also causes a rapid increase in stiffness through the evaporation of bitumen oils [2]. Therefore, the factors responsible for the overall difference between the dynamic viscosity states of bitumen 50/70 M after foaming were the bitumen ageing process and the presence of FT wax crystallites. Note that the phase transition temperature of synthetic wax is approximately 105 °C [16,38]. As mentioned in Section 4.2, a singularity for the combination of 100-110-1 was observed in both bitumen types. During the test of the dynamic viscosity η for the Newtonian state and the zero-shear dynamic viscosity η_ZSV_, the results for this combination were lower than for the non-foamed bitumen. The foaming process effect was marginal but a certain amount of water was trapped in the bitumen. The presence of the nucleins resulting from bitumen shearing results in rapid yielding. The marginal impact of the ageing process makes bitumen more susceptible to shear at this configuration, and can contribute to poor performance of bituminous mixtures made with this bitumen. The ageing index IS, used to determine the scale of bitumen ageing, was calculated as the ratio of the dynamic viscosity prior to foaming to that after foaming, with Equation (5):

(5) $$\;\mathrm{IS}=\frac{\eta_{60}^{\mathrm{foamed}}}{\eta_{60}^{\mathrm{non}-\mathrm{foamed}}}\;$$

where: IS is the ageing index, $\eta_{60}^{\mathrm{foamed}}$ is dynamic viscosity (Newtonian state) of bitumen foamed at 60 °C, and $\eta_{60}^{\mathrm{non}-\mathrm{foamed}}$ is dynamic viscosity (Newtonian state) of non-foamed bitumen at 60 °C.

In Poland, the ageing index of bitumen ranges from 1.5 (low stiffness increase after ageing) to 3.0, but should not be greater than 4.0 [39]. Table 7 summarises the results described by the regression model.

**Figure 9:**
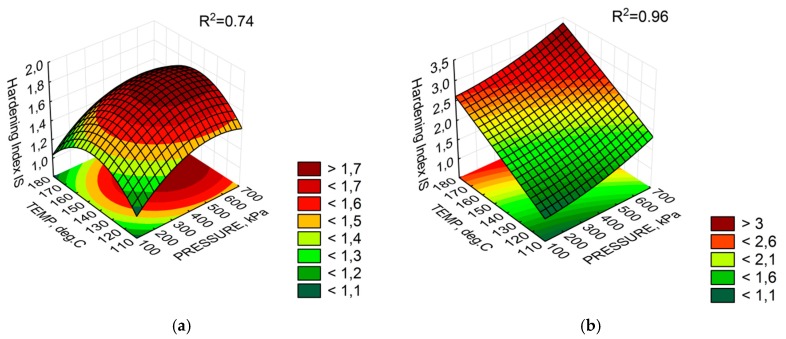
Response surface of the ageing index IS at 2.5% water: (**a**) bitumen 50/70; and (**b**) bitumen 50/70 M.

As in the evaluation of G*/sin(δ), the bitumen tested with the 100-110-1 configuration had a lower ageing index value than the base bitumen. This confirms the impact of trapped water on bitumen yielding. Analysis of the fit results in Table 6 shows that 50/70 M bitumen is ageing due to the influence of temperature and in particular water. The presence of synthetic wax enhances the IS increase. Bitumen 50/70 was affected by the temperature and water pressure. However, the small number of significant parameters confirms the low sensitivity of foamed 50/70 bitumen to ageing. The average error of estimation does not exceed 0.17 at the correlation coefficient of 0.74, which can be considered as a good fit of the experimental data to the approximated model of the function. It should be noted that regardless of the type of bitumen used, at least one foaming parameter had a significant effect on the ageing index model. This proves the hypothesis that changes in bitumen viscosity during foaming are not accidental and indicate a progressing ageing process. A graphic representation of the fit of the response surface model is shown in Figure 9.

The IS results shown in Figure 8 have a similar distribution to that of G*/sin(δ) and confirm the hypothesis that the foaming process significantly alters rheological properties of bituminous material. In this case, high temperature and pressure increase the value of IS in bitumen 50/70 M. With a given amount of water, the bitumen temperature of 180 °C and the pressure of 700 Pa, the ageing index reached the value close to 4.0. For bitumen 50/70, the IS variability is lower than that of 50/70 M and reaches a maximum of 1.79 for the 547-145-2.8 configuration. It should be noted that the variation in the hardness index for bitumen 50/70 is small compared to 50/70 M. The presence of synthetic wax intensifies the increase in the IS value. Based on these observations, consideration should be given to including the ageing phenomenon as a factor in the selection of bitumens for foaming. It is important to find the optimal settings of the WLB-10S with regard to the distribution of foaming parameters and to analyse rheological performance characteristics. The results of these analyses are of key importance for the technological process during which foaming parameters determine adequate aggregate coating while maintaining the appropriate stiffness needed for the durability of pavement layers.

## 5. Optimisation Process

**Figure 10:**
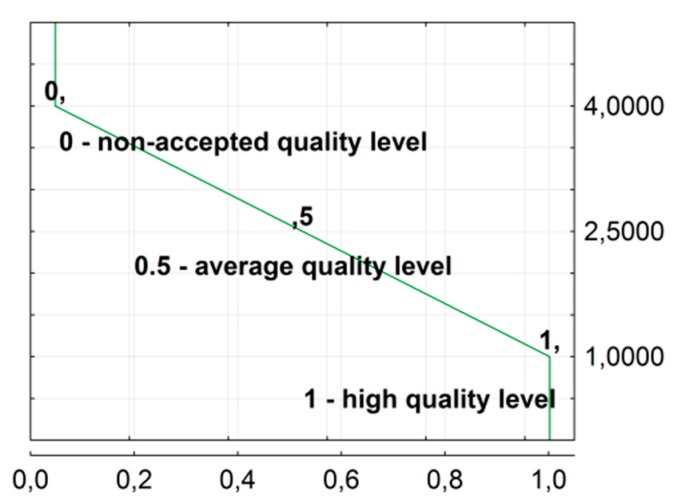
Example of a utility profile based on the ageing index IS.

Results for foaming confirm the observations reported in the literature that the correlation between the ER and HL parameters is not determined conclusively [7,40]. The bitumen rheological properties, i.e., dynamic viscosity and stiffness modulus, change during foaming. This makes it unclear where the optimum of foaming parameters is, because, for example, the settings at which high quality bitumen foam can be obtained coincide with a change in bitumen stiffness. To resolve the problem of how to obtain the maximum efficiency of foaming, the optimisation process was applied to simultaneously maximise input data by maximising the utility function. The optimisation function, which is in fact the geometric mean of partial utilities, was used based on the utility profiles. Detailed theoretical information can be found in [25,26,41]. First, it was necessary to determine the utility profile, which transforms each target variable to a value from the range <0;1>, thus expressing satisfaction with the result obtained at a given level. In fact, there is a range of acceptable values for the target variable. The analysis used a linear utility profile between the lowest and highest satisfaction with the results. The example profile for the IS variable is shown in Figure 10.

Optimisation has an infinite number of solutions. The desirable result is determined by the adopted criteria. Table 8 summarises target variable ranges required for establishing utility profile ranges and gives relevant literature references. 

**Figure 11:**
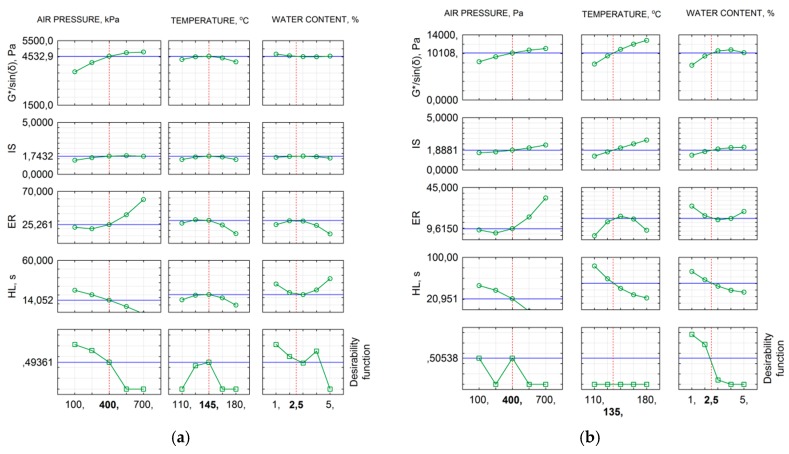
Optimisation outcomes: (**a**) bitumen 50/70; and (**b**) bitumen 50/70 M (2.5% FT wax).

The main goal of the optimisation was to find a combination of the WLB-10S settings with which the value of the generalised utility function would be about 0.5. This value would guarantee a moderately satisfactory result with all the requirements met (Table 8). The profiles of approximated solutions search with utility profiles accounted for, in the Statistica package, are shown in Figure 11.

The search for the optimal solution for a utility function of about 0.5 was performed using the simplex method. With regard to bitumen 50/70, the 400-145-2.5 configuration is the optimum configuration of foaming parameters assuming that the requirements for the foam quality distribution parameters and the ageing index are met. Considering the utility profiles obtained, more water, up to 3.0%, can be used. This will, however, increase the ageing index. Obtaining the optimum for bitumen 50/70 M with 2.5% synthetic wax is burdened with lower tolerance and a moderately satisfactory result is possible at a temperature 10 °C lower than in bitumen 50/70. As an optimal solution, it is understood to achieve average and acceptable bitumen foam performance (desirability function equal to 0.5). The optimal configuration of the foaming process parameters is 400-135-2.5. This will be of great importance from the perspective of technological processes of bituminous mix manufacture with such bitumen. It should be noted that the change in bitumen composition is very important from the perspective of bitumen rheological properties and rheology if the bitumen foam. Regardless of the type of bitumen, the increase in water volume and temperature at high pressures results in increased ER leading to rapid bitumen ageing and a reduction in the HL of the foam. Table 9 compiles the results obtained with the established optimal settings of the laboratory-scale bitumen foamed plant.

From the characteristics of the tested bitumens shown in Table 9, it follows that bitumen 50/70 reached better and higher ER values than bitumen 50/70 M. The ageing factor is comparable in both bitumen types. Bitumen 50/70 M had the ER value higher than minimum with a considerably higher HL, indicating a more stable foam in this bitumen. Despite lower ER, G*/sin(δ) in bitumen 50/70 M was 2.23 times that of bitumen 50/70. Considering the effect of bitumen 50/70 M incorporated in H-WMA mixtures in terms of the resistance to permanent deformation, the results are good unless severe bitumen ageing is entailed. To increase ER in bitumen 50/70 M, the temperature needs to be increased with a slight increase in pressure and water amount applied. This will result in bitumen stiffness increase due to ageing and effective HL reduction. This study demonstrates that ageing of bitumen 50/70 and 50/70 M can be limited with acceptable bitumen properties maintained. Referring to deep cold recycling technology, an increase in bitumen stiffness will also significantly affect the stiffness modulus of the recycled layer, as also mentioned in [37]. As a result, the use of synthetic wax in bitumen 50/70 will ensure that the bitumen foam will have acceptable ER and will be stable, with a longer period in which a potential bituminous mixture will be workable and, ultimately, will have higher stiffness in service. Nevertheless, this method and optimisation results have some limitations:

- The basic limitation of this method is the lack of validation in terms of reproducibility. However, repeatability results make this method promising and more accurate in comparison with common calibrated rod method.
- A 3^(3−1)^ experimental design does not take into account the interaction. Therefore, additional explanation of the model variability, coming from interactions, was not possible.
- The model used for determining ER and HL took into consideration only neat bitumen 50/70 penetration grade. Certain singularities associated with the foaming process for other bitumens with a different penetration grade may be omitted. For this reason, more sophisticated mathematical models would be needed. Such research and analyses are still ongoing.

## 6. Conclusions

The following conclusions were drawn from the tests for foaming optimisation in the context of bitumen viscoelastic performance changes:

- Digital registration of ER and HL values improves the accuracy of the measurement and minimises the impact on the part of operators. The indicative increase in measurement accuracy is six times that of the classical approach.
- Comparative analysis of foam distribution function parameters indicates that the presence of FT wax stabilises the foam decay rate over time.
- The G*/sin(δ) parameter is particularly sensitive to bitumen temperature and air pressure changes. Regardless of the bitumen type, G*/sin(δ) increased considerably after foaming, compared to the non-foamed reference bitumen.
- Significant differences were observed between dynamic viscosity and zero shear viscosity at the test temperature of 60 °C. The largest differences were found in bitumen 50/70 M due to the presence of crystallites of the synthetic wax.
- The foaming process causes a horizontal shift of the Black curve to lower values of phase angle δ. The same characteristics are exhibited by road bitumen subjected to simulated ageing.
- The significance of the adopted mathematical models of bitumen rheological performance indicates that foaming strongly affects rheological parameters of the control bitumen.
- Evaluation of the increase in stiffness of the bitumen after foaming, using the ageing index, revealed that foaming increases bitumen viscosity. This suggests that the foaming process distillates the oil fractions from the bitumen. The process intensifies with increasing temperature, water amount and air pressure.
- A certain singularity was identified in bitumen behaviour for the 100-110-1.0 combination, regardless of the bitumen type. For a given combination of the foaming apparatus settings, a stiffness decline was recorded, attributed to the presence of air bubbles in the bitumen, which at the negligible impact of ageing caused an increase in bitumen susceptibility to shear.
- The fractional experimental design considerably facilitates the optimisation of foaming parameters. Optimisation in terms of water alone does not guarantee good results. The findings in this study reveal that the foam distribution changes noticeably also with air pressure and temperature change, which may lead to an adjustment of the amount of water during foaming.
- The best foaming parameters for bitumen 50/70 were obtained for the 400-145-2.5 combination. The best combination for bitumen 50/70 M was 400-135-2.5.
- The optimisation results at the adopted utility function level of about 0.5 indicate that bitumen 50/70 M had a lower but acceptable expansion compared to bitumen 50/70. Bitumen 50/70 M had a longer and more stable foam decay rate and G*/sin(δ) that was 2.23 times higher than that for bitumen 50/70. This level of stiffness will have a beneficial effect on the resistance to permanent deformations in the mixtures produced with this bitumen. These results were obtained for the low ageing index in the range 1.7–1.9.

## Figures and Tables

**Table 1:** Bitumen test results.

Parameter	u·m	Standard	Results	
			50/70	50/70 M (2.5% FT wax)
Penetration	0.1 mm	PN-EN 1426	65	58
Softening point	°C	PN-EN 1427	49	73
G*/sinδ at 60 °C and 1.59 Hz	Pa	PN-EN 14770	4093	5483
η at 60 °C	Pa·s	PN-EN 12596	350	339
η_ZSV_ at 60 °C	Pa·s	CEN/TS 15325	542	656

**Table 2:** Experimental design 3^3−1^.

Standard Run	3^(3−1)^ Fractional Factorial Design, 1 Block, 9 Runs		
	Air Pressure, kPa	Temperature, °C	Water Proportion, %
1	100	110	1
4	400	110	5
7	700	110	2.5
2	100	145	5
5	400	145	2.5
8	700	145	1
3	100	180	2.5
6	400	180	1
9	700	180	5

**Table 3:** Initial comparative evaluation of uncertainties of foamed bitumen ER measurement methods.

Method	Source of Uncertainty	Distribution of Measurands	Partial Uncertainty
**Original Method (prototype)**	Sensor resolution (±5 µm at the measured distance of 50 cm)	rectangular	0.001 mm
	Accuracy class (0.05% measured quantity + 0.01% measurement range)	rectangular	0.104 mm
	Sensor repeatability of measurement (±15 µm at the measured distance of 50 cm)	rectangular	0.009 mm
	Linearity error (±20 µm at the measured distance of 50 cm)	rectangular	0.004 mm
	Amplitude of readings due to measured sample surface irregularity based on the max RMSE of the estimated regression functions (read for 700-110-2.5)	normal	1.87 mm
	**Expanded Uncertainty (k = 2) of ER** **U(ER) = 0.51**		
**Classical Method**	Measuring rod resolution (every 5 ER units in the calibrated container)	rectangular	1.44 (ER)
	**Expanded Uncertainty (k = 2) of ER** **U(ER) = 2.9**		

**Table 4:** Distribution of foaming parameters.

CASE	Bitumen Type									
	50/70 (a = 0.6 cm)					50/70 M (a = 0.6 cm)				
	b_50/70_, cm	HL_50/70_, s	c_50/70_	RMSE(b_50/70_), cm	R^2^	b_50/70M_, cm	HL_50/70M_, s	c_50/70M_	RMSE(b_50/70M_), cm	R^2^
100-110-1	1.86	34.25	2.74	0.48	0.99	1.58	135.22	1.10	0.28	0.97
100-145-5	3.33	56.13	1.54	1.38	0.98	4.80	20.48	0.94	0.97	0.99
100-180-2.5	3.48	0.001	0.09	0.91	0.61	35.00	0.002	0.45	1.50	0.65
400-110-5	2.82	16.99	1.16	0.56	0.98	3.56	44.79	1.66	1.19	0.98
400-145-2.5	11.18	16.14	1.50	1.58	0.98	5.77	20.95	2.32	1.40	0.98
400-145-2.5 (additional)	12.42	15.05	1.50	1.68	0.98	5.62	22.41	2.21	1.65	0.99
400-145-2.5 (additional)	11.01	15.89	1.53	1.84	0.97	5.52	19.02	2.32	1.45	0.98
400-180-1	1.10	123.00	0.90	1.04	0.78	1.02	14.08	4.37	0.40	0.65
700-110-2.5	35.0	0.20	0.82	1.54	0.93	4.68	20.37	1.48	1.87	0.97
700-145-1	35.0	1.28	0.88	1.42	0.98	35.00	0.78	0.63	1.50	0.98
700-180-5	5.89	7.20	3.10	0.98	0.87	7.03	10.00	2.25	0.46	0.99

**Table 5:** Fitting parameters of the objective function of foam distribution parameters (ER and HL) for bitumen 50/70 and bitumen 50/70 M (2.5% FT wax) with respect to Equation (1).

Regression Model Parameters	Bitumen Type							
	Bitumen 50/70				Bitumen 50/70 M (2.5% FT wax)			
	Variable—ER R^2^ = 0.92 RMSE(ER) = 4.0		Variable—HL R^2^ = 0.73 RMSE(HL) = 5.8 s		Variable—ER R^2^ = 0.83 RMSE(ER) = 1.3		Variable—HL R^2^ = 0.98 RMSE(HL) = 8.9 s	
	Coefficient	*p*-Value	Coefficient	*p*-Value	Coefficient	*p*-Value	Coefficient	*p*-Value
Mean/Const.	−168.356	0.001611	**−107.349**	**0.080420**	−294.983	<0.0001	638.2769	0.000074
(1) PRESSURE (L **)	−0.072	0.003419	**−0.026**	**0.350907**	−0.066	<0.0001	**−0.0022**	**0.960100**
PRESSURE (Q ***)	0.000	<0.0001	**−0.000**	**0.466805**	0.000	<0.0001	−0.0002	0.017506
(2) TEMP (L)	2.670	0.000712	2.450	0.010745	4.632	<0.0001	−6.0151	0.001159
TEMP (Q)	−0.010	0.000366	−0.009	0.008153	−0.016	<0.0001	0.0159	0.005498
(3) WATER (L)	15.446	0.001344	−25.045	0.000603	−20.245	<0.0001	−35.7525	0.001552
WATER (Q)	−3.174	0.000239	4.477	0.000277	3.118	<0.0001	3.3096	0.031701

* the values given in bold are not statistically significant; ** linear component (L); *** quadratic component (Q).

**Table 6:** Fitting parameters of the objective function of G*/sin(δ) for bitumen 50/70 and 50/70 M (2.5% FT wax).

Regression Model Parameters	Bitumen Type			
	Bitumen 50/70		Bitumen 50/70 M (2.5% FT Wax)	
	Regression Coefficients R^2^ = 0.72 RMSE(G*/sin(δ)) = 137.3 Pa		Regression Coefficients R^2^ = 0.99 RMSE(G*/sin(δ)) = 128.3 Pa	
	Coefficient	*p*-Value	Coefficient	*p*-Value
Mean/Const.	−935.887 **	0.823416 **	−18160.0	0.000000
(1) PRESSURE (L)	5.165	0.024038	8.8	0.000000
PRESSURE (Q)	−0.004 **	0.134533 **	−0.001	0.000132
(2) TEMP (L)	61.657 **	0.305960 **	214.8	0.000001
TEMP (Q)	−0.220	0.0485013	−0.5	0.000022
(3) WATER (L)	−177.080 **	0.637377 **	3270.2	0.000000
WATER (Q)	24.229 **	0.686761 **	−432.0	0.000000

** the values given in bold are not statistically significant.

**Table 7:** Fitting parameters of the objective function of IS for bitumen 50/70 and bitumen 50/70 M (2.5% FT wax).

Regression Model Parameters	Bitumen Type			
	Bitumen 50/70		Bitumen 50/70 M (2.5 FT Wax)	
	Variable IS R^2^ = 0.74 RMSE(IS) = 0.17		Variable IS R^2^ = 0.96 RMSE(IS) = 0.14	
	Coefficient	*p*-Value	Coefficient	*p*-Value
Mean/Constant	−4.64698	0.020737	−2.49385 **	0.112468 **
(1) PRESSURE (L)	0.00240	0.014726	0.00014 **	0.840934 **
PRESSURE(Q)	0	0.053273 **	0	0.164181 **
(2) TEMP (L)	0.07574	0.009483	0.02506	0.048073
TEMP (Q)	−0.00026	0.008850	−0.00001 **	0.875894 **
(3) WATER (L)	0.21186 **	0.195377 **	0.49955	0.003225
WATER (Q)	−0.03816 **	0.149286 **	−0.05180	0.035854

** the values given in bold are not statistically significant.

**Table 8:** Limiting ranges of utility profiles.

Level	ER	HL, s	G*/sin(δ), Pa	IS
**Low (0)**	<8 [4,40,42]	≤12 [4,43,44]	<2200 [34,35]	≥4 [39]
**High (1)**	>15 [4,45]	≥45 [4,45]	≥2200 [34,35]	<1.5 [39]

**Table 9:** Comparison of bitumen foam optimisation results with regard to the DOE.

Parameter	Bitumen Type	
	Bitumen 50/70	Bitumen 50/70 M
Optimisation Outcome	400 kPa-145 °C-2.5%	400 kPa-135 °C-2.5%
ER	25.3	9.6
HL, s	14.1	21
G*/sin(δ), Pa	4533	10108
IS	1.7	1.9
